# Phase II randomised trial of raltitrexed–oxaliplatin *vs* raltitrexed–irinotecan as first-line treatment in advanced colorectal cancer

**DOI:** 10.1038/sj.bjc.6602860

**Published:** 2005-11-01

**Authors:** J Feliu, C Castañón, A Salud, J R Mel, P Escudero, A Pelegrín, L López-Gómez, M Ruiz, E González, F Juárez, J Lizón, J Castro, M González-Barón

**Affiliations:** 1 Service of Medical Oncology, La Paz, Paseo de la Castellana 261, Madrid 28046, Spain; 2 Service of Medical Oncology, Complejo Hospitalario de León, C/ Altos de Nava s/n, León, 24071, Spain; 3 Service of Medical Oncology, H Arnau de Villanova, Avda. Alcalde Roure 80, Lérida 25006, Spain; 4 Service of Medical Oncology, H Xeral, Doctor Severo Ochoa s/n, Lugo 27004, Spain; 5 Service of Medical Oncology, H Lozano Blesa C/ San Juan Bosco 15, Zaragoza, 50009, Spain; 6 Service of Medical Oncology, Virgen de la Salud, Avda. Barber 35, Toledo 45004, Spain; 7 Service of Medical Oncology, H Punta de Europa, Ctra. De Getares s/n, Algeciras 11207, Spain; 8 Service of Medical Oncology, H Virgen de las Nieves, Avda. Coronel Muñoz, Granada 18012, Spain; 9 Service of Medical Oncology, H Nta. Señora del Prado, Ctra. De Madrid Km 114, Talavera 45600, Spain; 10 Service of Medical Oncology, H San Juan, Ctra. Alicante-Valencia s/n, Alicante 03550, Spain

**Keywords:** colorectal cancer, raltitrexed, oxaliplatin, irinotecan, toxicity

## Abstract

The purpose of this phase II randomised trial was to determine which of two schemes, raltitrexed-irinotecan or raltitrexed-oxaliplatin, offered better activity and less toxicity in patients with advanced colorectal cancer (CRC). A total of 94 patients with previously untreated metastatic CRC were included and randomised to receive raltitrexed 3 mg m^−2^ followed by oxaliplatin 130 mg m^−2^ on day 1 (arm A), or CPT-11 350 mg m^−2^ followed by raltitrexed 3 mg m^−2^ (arm B). In both arms treatment was repeated every 3 weeks. Intent-to-treat (ITT) analysis showed an overall response rate of 46% (95% CI, 29.5–57.7%) for arm A, and 34% (95% CI, 19.8–48.4%) for arm B. Median time to progression was 8.2 months for arm A and 8.8 months for arm B. After a median follow-up of 14 months, 69% of patients included in arm A were still alive, compared to 59% of those included in arm B. Overall, 31 patients (65%) experienced some episode of toxicity in arm A and 32 patients (70%) in arm B, usually grade 1–2. The most common toxicity was hepatic, with 29 patients (60%) in arm A and 24 patients (62%) in arm B, and was grade 3–4 in four (8%) and four (9%) patients, respectively. In all, 14 patients (29%) from arm A and 24 patients (52%) from arm B had some grade of diarrhoea (*P*<0.03). Neurologic toxicity was observed in 31 patients (64%) in arm A, and was grade 3–4 in five patients (10%), while a cholinergic syndrome was detected in nine patients (19%) in arm B. There were no differences in haematologic toxicity. One toxic death (2%) occurred in arm A and three (6.5%) in arm B. In conclusion, both schemes have high efficacy as first-line treatment in metastatic CRC and their total toxicity levels are similar. Regimens with raltitrexed seem a reasonable alternative to fluoropyrimidines.

For more than 40 years, 5-fluorouracil (5FU) was the only cytotoxic agent with significant activity in advanced colorectal cancer (CRC). Recently, however, the topoisomerase I inhibitor irinotecan (CPT-11) and the platinum compound oxaliplatin have shown efficacy as second-line single-agent therapy and as first-line therapy in combination with fluoropyrimidines. In large phase III trials, irinotecan in combination with 5FU/leucovorin (LV) and oxaliplatin in combination with 5FU/LV showed superior efficacy compared with 5FU/LV alone as first-line therapy (Douillard *et al*, 2000; Giacchetti *et al*, 2000; de Gramont *et al*, 2000; Saltz *et al*, 2000; Grothey *et al*, 2002).

Although combined therapy has been an important development in the treatment of advanced CRC, these regimens do have some disadvantages. Firstly, their increased toxicity, particularly with the CPT-11–5FU–LV combination when 5FU is administered by bolus (Ledermann *et al*, 2001; Rothenberg *et al*, 2001). Secondly, continuous-infusion-based regimens require the use of implantable access devices and portable infusion pump, which is associated with an increased risk of infection and thromboembolism (Clark and Raffin, 1990; Prandoni and Bernardi, 1999; Verso and Agnelli, 2003). Thirdly, the inconvenience and discomfort of regular hospital visits for intravenous (i.v.) drug administration, which can have a negative effect on their quality of life.

Raltitrexed is a specific inhibitor of thymidylate synthase. This enzyme has a fundamental role in the *de novo* synthesis of the nucleotide thymidine triphosphate, which is essential for DNA synthesis. At a dose of 3 mg m^−2^, it is active in a variety of tumours such as breast, pancreatic or refractory ovarian cancers (Cunningham *et al*, 1994), but it is in colorectal tumours where it shows the highest activity (Van Custem *et al*, 2002). Several phase III studies performed in patients with advanced CRC demonstrated that response rates and survival were similar to that of the combination 5FU–LV (Cunningham *et al*, 1995; Cocconi *et al*, 1998; Maughan *et al*, 2002). However, in another randomised trial, a survival advantage in favour of patients treated with conventional 5FU–LV was seen even when the response rate was similar (Pazdur and Vicent, 1997).

Administration as a short 15-min i.v. infusion every 3 weeks adds value to the efficacy and toxicity profile of raltitrexed. In fact, in a study which compared the patients' preferences between raltitrexed and other 5FU-based regimens with regard to side-effects and administration attributes, 91% of the patients expressed a preference for the former treatment (Young *et al*, 1999).

Several studies have shown a sequence-specific synergistic cytotoxicity for the combination of raltitrexed with CPT-11 (Aschele *et al*, 1988), while for the combination of raltitrexed with oxaliplatin both synergistic and additive antineoplastic activities have been observed (Raymond *et al*, 1997).

Several phase II studies with the raltitrexed–CPT-11 combination have shown response rates of 34–46% (Carnaghi *et al*, 2002; Feliu *et al*, 2004) and median survivals of 12–15.6 months. On the other hand, with the raltitrexed–oxaliplatin combination, response rates of 41–54% and median survivals of 14.6–14.8 months have been reported (Cascinu *et al*, 2002; Seitz *et al*, 2002; Santini *et al*, 2004). These results are comparable to those achieved with 5FU–LV combinations with CPT-11 or oxaliplatin.

In view of these encouraging clinical data and the continuing need for active regimens with an alternative mechanism of action other than 5FU–LV, evaluation of the therapeutic potential of the combination of raltitrexed with CPT-11 or oxaliplatin seems to be of considerable interest. We thus decided to initiate a randomised phase II study in previously untreated patients with advanced CRC to determine which of two schemes, raltitrexed–CPT-11 or raltitrexed–oxaliplatin, offered higher activity and less toxicity, with a view to their selection for a subsequent phase III trial which would compare this regimen with a control arm treated with a regimen of 5FU–LV and CPT-11 or oxaliplatin.

## PATIENTS AND METHODS

### Patient population

A total of 94 patients with recurrent or metastatic CRC were included from May 2002 to December 2003. They all had at least one lesion histologically confirmed adenocarcinoma. Patients who had received prior adjuvant 5FU-based chemotherapy were eligible if they had remained free of disease for at least 6 months after completion of the adjuvant therapy. Patients with operable metastatic disease were excluded from the study. Other inclusion criteria were: (1) a performance status ⩽2, according to the Eastern Cooperative Oncology Group (ECOG) scale; (2) life expectancy of at least 3 months; (3) adequate bone marrow function, that is, a granulocyte count ⩾2 × 10^9^ l^−1^ and platelets >100 × 10^9^ l^−1^; (4) peripheral neuropathy (⩽grade 1, National Cancer Institute Common Toxicity Criteria (NCI-CTC) scale); (5) adequate hepatic function, that is, serum bilirubin <1.25 times the upper normal limit, glutamic oxaloacetic transaminase values (SGOT) and glutamic pyruvic transaminases (SGPT) <2.5 times the upper normal limit in the absence of hepatic metastases or <5 times the upper normal limit in the presence of metastasis; (6) adequate renal function, that is, a creatinine value ⩽1.25 times the upper normal limit and creatinine clearance >65 ml min^−1^.

Patients with any prior chemotherapy for advanced disease, brain or meningeal metastases, or a history of any other malignancy, were excluded, except in cases of basal cell carcinoma or *in situ* cervical carcinoma adequately treated. Patients provided written informed consent according to the directives of the local ethical committees.

All patients had measurable disease, as defined by the presence of at least one unidimensionally measurable lesion by computed tomography scan. Pleural effusion, ascites, osteoblastic lesions or previously irradiated lesions were not accepted as measurable disease. Patients who had received radiotherapy were eligible if there was at least one measurable lesion outside the radiation field.

### Treatment plan

Chemotherapy consisted of 3-weekly courses either of raltitrexed 3 mg m^−2^ as a 15-min i.v. infusion followed 45 min later by oxaliplatin 130 mg m^−2^ on day 1, given as a 2-h infusion in 250 ml of dextrose 5% (arm A), or 3-weekly courses of CPT-11 350 mg m^−2^ in a 30-min i.v. infusion followed by a 15-min infusion of raltitrexed 3 mg m^−2^ (arm B). Antiemetic and symptomatic therapies were permitted, with the exception of any vitamin supplement containing folic acid. Patients were to be withdrawn from the study as a result of any of the following: (1) objective disease progression (according to protocol criteria); (2) unacceptable toxicity or adverse event; (3) patient unwilling or unable to continue (dropouts); (4) patient lost to follow-up; (5) investigator decision that it was in the patient's best interest not to continue.

Patients were assessed for toxicity before each course and graded according to NCI-CTC version 2. Therapy was delayed for 1 week if the neutrophil count was <1.5 × 10^9^ l^−1^ or the platelet count <100 × 10^9^ l^−1^, or for significantly persisting nonhaematological toxicity. Therapy was definitely discontinued if toxicity persisted after a 2-week delay. In case of grade 3 or 4 haematological toxicity, the dose of all drugs was decreased by 25 or 50%, respectively. If grade 2 or 3 diarrhoea or stomatitis occurred, the dose was reduced by 25 or 50%, respectively; grade 4 diarrhoea or stomatitis led to treatment withdrawal. The oxaliplatin dose was reduced by 25% for subsequent cycles in case of persistent (⩾14 days) or temporary (7–14 days) painful paresthesia or functional impairment. In case of persistent painful paresthesia or functional impairment, or if a patient experienced any other severe neurotoxicity despite a 25% dose attenuation, oxaliplatin was omitted in subsequent cycles. In case of the occurrence of a laryngeal spasm syndrome, the duration of the oxaliplatin infusion was increased from 2 to 6 h. In case of persistent problems, oxaliplatin was omitted. The Cockcroft–Gault formula (Cockcroft and Gault, 1976) was used to calculate creatinine clearance before each cycle. If creatinine clearance was between 55 and 65 ml min^−1^, the dose of raltitrexed was reduced by 25% and the next cycle given 4 weeks later. If it was between 25 and 54 ml min^−1^ the dose of raltitrexed was reduced by 50% and the next cycle given 4 weeks later, and if it was <25 ml min^−1^ the treatment was interrupted.

### Pretreatment and followup studies

A diagnostic workup was performed within 3 weeks prior to the start of treatment, consisting of a complete clinical history, physical examination, performance status assessment, haematological and biochemical profiles (including CEA level), a chest X-ray and a computed tomography scan of the chest and abdomen at baseline. Additional imaging investigations were performed if clinically indicated. A computed tomography scan was repeated every three courses to assess the objective response. At the end of chemotherapy, all clinical, laboratory and imaging studies were repeated and patients underwent followup examination every 2 or 3 months until death.

### Response criteria

Patients were evaluated clinically on an ITT basis at least every 3 weeks and radiographically every 9 weeks. The same evaluation modality was used throughout the study. RECIST response guidelines were used (Therasse *et al*, 2000), defining all responses after at least 9 weeks of therapy as follows: complete response (CR), partial response (PR), stable disease (SD) or progressive disease (PD). We defined disease control as the sum of patients achieving a CR, PR or SD. Confirmation of all responses was required after 4 weeks. Duration of response was defined as the period from the first day the criteria for CR or PR were met to the progression date. Time to tumour progression (TTP) was estimated from the dates of the first course of treatment to the first documentation of disease progression. Survival was calculated by the same method from the date of the first cycle of treatment until the date of death or last known followup.

### Statistical analysis

The primary end point was response rate and the secondary objectives time to progression and toxicity. Dose intensity was calculated by dividing the total mg m^−2^ of drug given by the number of weeks elapsed from the beginning of therapy to the end of the last cycle.

The ‘pick the winner’ format based on the randomised phase II clinical trials approach as proposed by Simon *et al* (1985) was used. In this approach, an accrual of 38 patients in each arm gives a 90% chance of selecting the better treatment schedule if the difference in response rate is at least 15% and the smaller response rate is assumed to be approximately 35%. Also, 10% was added to this figure to allow for losses. If both schedules were between the pre-established ranges of response rate, the less toxic regimen would be chosen.

The Wilcoxon rank-sum method was used to compare quantitative variables, the Fisher's exact test for percentages, and the Kaplan–Meier method for survival, TTP and the duration of response.

## RESULTS

### Patient characteristics

A total of 94 patients with recurrent or metastatic CRC were entered into the study and received the allocated treatment subsequent to randomisation. In all, 48 patients were randomised to arm A (oxaliplatin/raltitrexed) and 46 to arm B (irinotecan/raltitrexed). As shown in Table 1, the two groups were well matched for pretreatment characteristics. The patients' median age was 64 years, and the majority (95%) had an ECOG performance status of 0 or 1. The primary tumour was located in the colon in 58 (62%) and in the rectum in 36 (38%). Synchronous metastatic disease was observed in 48 patients (51%), in 22 of which (23%) the primary tumour was not resected. Of the remaining patients, 46 (50%) presented metastases secondary to a tumour previously removed. Of these patients, 31 (33%) had previously received adjuvant chemotherapy and 11 (12%) had received chemotherapy and radiotherapy. The liver was the predominant metastatic site (68%), and the median number of involved sites was two per patient.

Seven patients were unassessable for treatment efficacy, four in arm A (one because of severe toxicity, one due to toxic death, one moved to a different city and one due to death apparently unrelated to the neoplasia, i.e., acute myocardial infarction) and three in arm B (two due to toxic death and one because of severe toxicity). These patients were retained for the ITT analysis.

### Dose intensities

Patients in arm A (oxaliplatin/raltitrexed) received a total of 303 cycles, with a median of six cycles per patient (range 1–16). Patients in arm B (irinotecan/raltitrexed) received 286 cycles, with a median of six cycles per patient (range 1–23). In seven patients (15%) from arm A and eight (17%) from arm B a delay occurred in administration of the treatment. In arm A the reasons for the delay were: elevation of transaminases in four, diarrhoea in two and thrombopenia in one, and in arm B elevation of transaminases in four, diarrhoea in two and neutropenia in two. In arm A, the median dose intensity was 41 mg m^−2^ week^−1^ for oxaliplatin and 0.95 mg m^−2^ week^−1^ for raltitrexed. These doses represent 95% of the scheduled doses both for oxaliplatin and raltitrexed. In arm B, the median dose intensity was 114 mg m^−2^ week^−1^ for irinotecan and 0.97 mg m^−2^ week^−1^ for raltitrexed. These doses represent 98% of the scheduled dose for irinotecan and 97% of the scheduled dose for raltitrexed.

### Tumour response and survival

The data for response, progression-free survival and overall survival are shown in Table 2. Assessment of response was by ITT on the total population included (48 patients in arm A and 46 in arm B) and per protocol on the patients who received three or more treatment cycles and were fully assessable for response (46 in arm A and 43 in arm B). According to the ITT analysis, the overall response rate was 46% (95% CI 29.5–57.7%) for arm A (oxaliplatin/raltitrexed) and 34% (95% CI, 19.8–48.4%) for arm B (irinotecan/raltitrexed). These differences were not significant (*P*>0.05). In the per protocol analysis, the overall response rates were 49% (95% CI, 33.3–62.9%) and 37% (95% CI, 21.2–51.3%) (*P*>0.05), respectively. Median duration of response was 7.9 and 9.2 months, respectively (*P*=0.696, log-rank test). Overall, control of disease (CR+PR+SD) was achieved in 35 patients (69%) in arm A and in 31 patients (67%) in arm B.

At the time of performing the analysis, 37 patients (77%) in arm A and 34 patients (74%) in arm B had progressed. The median TTP was 8.2 months for arm A and 8.8 months for arm B (*P*=0.565, log-rank test). After a median follow-up of 14 months, 69% of the patients included in arm A were still alive, compared to 59% of those included in arm B, and therefore median survival could not be calculated.

Second-line chemotherapy with irinotecan was administered to 24 (65%) of 37 patients who progressed in arm A and second-line treatment with oxaliplatin to 21 (68%) patients who progressed in arm B.

### Toxicity

Overall, 31 patients (65%) experienced some toxicity in arm A and 32 patients (70%) in arm B, usually grade 1–2. The main toxicities were gastrointestinal and haematologic. Treatment-related side effects are shown in Table 3. In general, both regimens were well-tolerated. The most common toxicity was hepatic, with elevation of transaminases being detected in 29 patients (60%) in arm A and 24 patients (62%) in arm B, although grade 3–4 toxicity was only observed in four (8%) and four (9%) of patients, respectively. It should be noted that 14 patients (31%) from arm A had some grade of diarrhoea, while it was observed in 24 patients (52%) from arm B (*P*<0.05). These differences can be attributed to a higher frequency of grade 1–2 diarrhoea in arm B (23 *vs* 39%). However, the percentage of patients with grade 3–4 diarrhoea was similar in both groups: four patients (8%) in arm A and four patients (13%) in arm B. Another very common toxicity was asthenia, usually grade 1–2. Overall, asthenia was detected in 17 patients (35%) in arm A and in 24 patients (52%) in arm B. However, these differences were not significant from a statistical point of view (*P*=NS). In addition, neurologic toxicity was observed in 31 patients (64%) in arm A, and was grade 3–4 in five patients (10%), while a cholinergic syndrome was detected in nine patients (19%) in arm B. One toxic death (2%) occurred in arm A, due to diarrhoea, mucositis, neutropenia and sepsis, and three (6.5%) in arm B, two due to diarrhoea, neutropenia and sepsis and one due to diarrhoea, mucositis and acute renal failure.

## DISCUSSION

In recent years, the incorporation of new drugs active against CRC with different mechanisms of action has considerably expanded the therapeutic options. However, advanced CRC continues to be an incurable disease. Therefore, the objectives of treatment should be to prolong survival and alleviate symptoms, and to improve or at least maintain the quality of life of the patients. These objectives should be achieved by administering regimens that have low toxicity, are convenient and do not require repeated visits to the hospital by the patient, so as not to worsen the patient's quality of life. The primary objective of this phase II randomised trial was to determine the activity and tolerance of two chemotherapy schemes in first-line treatment of metastatic CRC, whose main advantage over bimonthly schemes is to reduce the number of hospital visits.

The results obtained in the study suggest that both schemes have similar efficacy, with no differences being detected in either response rate (46 *vs* 34%) or TTP (8.2 *vs* 8.8 months). The response rates achieved are similar to those reported by other authors, both with schemes combining raltitrexed with oxaliplatin (39–54% response rate) (Cascinu *et al*, 2002; Seitz *et al*, 2002; Santini *et al*, 2004; Grávalos *et al*, 2005) and those combining raltitrexed with irinotecan (34–46%) (Carnaghi *et al*, 2002; Feliu *et al*, 2004). Similarly, these results are comparable to those obtained with combinations of 5FU–LV with oxaliplatin or irinotecan (Douillard *et al*, 2000; Giacchetti *et al*, 2000; de Gramont *et al*, 2000; Saltz *et al*, 2000; Grothey *et al*, 2002; Tournigand *et al*, 2004). Although it is too soon to analyse the effects on survival, there will probably not be any significant differences, since, as pointed out by several authors, what is important is to use standard drugs throughout the disease, regardless of the order in which they are used (Grothey *et al*, 2004; Tournigand *et al*, 2004), and, in our study, 64% of the patients in the raltitrexed–oxaliplatin arm and 68% of those in the raltitrexed–irinotecan arm received a second-line treatment.

As expected, the two schemes showed some differences with regard to toxicity. The raltitrexed–irinotecan scheme was associated more frequently with diarrhoea, usually grade 1–2. In addition, a higher frequency of alopecia and asthenia was observed, although the latter did not achieve statistical significance. On the other hand, the raltitrexed–oxaliplatin scheme caused neurologic toxicity in 64% of patients, although it was grade 3–4 in only 10%. However, we should note the frequency of toxic deaths observed in the study, one in the raltitrexed–oxaliplatin arm (2%) and three (6.5%) in the raltitrexed–irinotecan arm, which represents an overall rate of toxic deaths of 4.1% in the study. These results contrast with our previous experience with the raltitrexed–irinotecan scheme in a phase II trial including 91 patients in which we did not observe any toxic death (Feliu *et al*, 2004). On the other hand, the toxic death rate with the bimonthly continuous-infusion regimens of 5FU–LV with either oxaliplatin or irinotecan is usually <3% (Douillard *et al*, 2000; Giacchetti *et al*, 2000; de Gramont *et al*, 2000; Goldberg *et al*, 2004; Tournigand *et al*, 2004). However, in the joint analysis of three phase III trials comparing raltitrexed monotherapy *vs* 5FU–LV, a 3.8% rate of toxic deaths was observed, although 65% of these deaths can be attributed to a protocol deviation (Zalckberg, 1997). In another more recent study, which also compared raltitrexed monotherapy with other 5FU–LV schemes, a 6% rate of toxic deaths was reported in the raltitrexed arm, although in some cases treatment was administered before the patients had completely recovered from a previous toxicity (Maughan *et al*, 2002). In this regard, special attention should be given to the renal function of the patients before each cycle of raltitrexed to avoid the occurrence of unexpected toxicities (Jansman *et al*, 2000; Van Custem *et al*, 2002). In fact, the AUC of raltitrexed can double when creatinine clearance is less than 65 ml min^−1^ (Judson *et al*, 1998). Nevertheless, despite the fact that in our study this aspect was contemplated in the protocol and it was stressed to investigators that they should take it into account, these toxic deaths still occurred. In this regard, it has also been pointed out that patients with low serum concentrations of folate have a higher risk of toxicity from raltitrexed (Keller *et al*, 2001). This would be due to cells taking up and retaining raltitrexed more avidly. We do not know to what extent this aspect may have played a role in the toxicity observed in our patients.

The main advantage of combinations with raltitrexed over bimonthly schemes is the possibility of reducing the number of hospital visits. In fact, in a phase II randomised trial comparing administration of raltitrexed–oxaliplatin with the FOLFOX4 scheme, a 50% reduction was observed in the number of hospital visits with the raltitrexed regimen (Grávalos *et al*, 2005). Although this can currently be achieved with schemes combining oral fluoropyrimidines with oxaliplatin or irinotecan (Cassidy *et al*, 2004), with these regimens frequent contacts with the treatment team are required to adjust the dose or temporarily interrupt treatment in order to avoid excessive toxicity.

Based on the above, although schemes with raltitrexed have similar efficacy as that observed with 5FU–LV combinations, due to its unpredictable toxicity and the development of regimens with oral fluoropyrimidines, interest in raltitrexed has declined substantially at present. Nevertheless, it could be useful in patients in whom the use of fluoropyrimidines is contraindicated for any reason such as ischaemic heart disease. In these situations, due to its lower toxicity, administration of the combination of raltitrexed–oxaliplatin could be considered.

## Figures and Tables

**Table 1:** Patients' characteristics

	**Oxaliplatin/raltitrexed**		**Irinotecan/raltitrexed**	
**Characteristic**	**No.**	**%**	**No.**	**%**
Age (mean and range)	63 (37–74)	65 (36–76)		
				
*Sex*				
Male	28	58	31	67
Female	20	42	15	33
				
*ECOG performance status*				
0	26	54	23	50
1	18	38	22	48
2	4	8	1	2
				
*Primary site*				
Colon	32	67	26	57
Rectum	16	33	20	43
				
*Metastasis sites*				
Liver	34	71	30	65
Lung	9	18	16	35
Abdominopelvic mass	18	37	13	28
Others	12	25	18	39
				
*No of metastatic sites*				
Single	19	40	17	37
Multiple	29	60	29	63
				
*Prior adjuvant therapy*				
None	27	56	24	52
Chemotherapy	14	29	17	37
Chemotherapy+radiotherapy	7	15	5	11

**Table 2:** Therapeutic results

	**Oxaliplatin/raltitrexed**		**Irinotecan/raltitrexed**	
**Results**	**No.**	**%**	**No.**	**%**
Complete response	4	8	2	4
Partial response	18	38	14	30
Stable disease	12	25	15	33
Progressive disease	11	23	12	33
Nonevaluables	3	6	3	7
Overall response rate (%) (95% confidence interval)	46% (29.5–57.7%)		34% (19.8–48.4%)	
		
*Response duration (months)*				
Median	7.9		9.2	
Range	5.4–9.5		6.3–10	
		
*Progression-free survival (months)*				
Median	8.2		8.8	
Range	5.8–10.4		6.8–10.6	

**Table 3:** Toxicity per patient according to NCI-CTC grade

	**Oxaliplatin/raltitrexed (*n*=48)**		**Irinotecan/raltitrexed (*n*=46)**	
**Toxicity**	**Grade 1–2**	**Grade 3–4**	**Grade 1–2**	**Grade 3–4**
Neutropenia	3 (6%)	1 (2%)	5 (11%)	4 (8%)
Anaemia	8 (17%)	2 (4%)	11 (24%)	1 (2%)
Thrombocytopenia	4 (8%)	2 (4%)	3 (7%)	2 (4%)
Nausea/vomiting	20 (42%)	6 (12%)	20 (43%)	3 (7%)
Diarrhoea	11 (23%)	4 (8%)	18 (39%)	6 (13%)
Stomatitis	3 (6%)	—	3 (7%)	2 (4%)
Transaminases	25 (53%)	4 (8%)	19 (53%)	4 (9%)
Asthenia	15 (31%)	2 (4%)	21 (46%)	3 (7%)
Peripheral neuropathy	26 (54%)	5 (10%)	—	—
Alopecia	2 (4%)	—	13 (28%)	2 (4%)
Cholinergic syndrome	—	—	9 (19%)	
